# Vinyl Ether Maleic Anhydride Copolymers: Efficient and Reusable Sorbents for Removing Heavy Metals from Water

**DOI:** 10.1021/acsmacrolett.6c00265

**Published:** 2026-07-02

**Authors:** Ranjita Thapa Acharya, Muhammad Zeeshan Shah, Audrey Woodruff, Dominik Konkolewicz

**Affiliations:** Department of Chemistry and Biochemistry, 6403 Miami University, 651 E. High St., Oxford, Ohio 45056, United States

## Abstract

Vinyl ethermaleic anhydride (VEMA) copolymers were synthesized by reversible addition–fragmentation chain transfer (RAFT) copolymerization and hydrolyzed to generate dicarboxylate-functionalized adsorbents for heavy metal removal from water. The RAFT-synthesized copolymer had superior removal efficiencies compared to a conventional free-radical synthesized analogue, while maintaining adsorption performance over multiple cycles. The metals could be removed under batch and flow conditions. Competitive experiments demonstrated adsorption in the order: Cu­(II) > Fe­(III) > Ni­(II) > Co­(II) > Zn­(II). The impact of pH and metal concentration was investigated. Overall, hydrolyzed VEMA copolymers are promising reusable adsorbents in water treatment applications.

## Graphical abstract

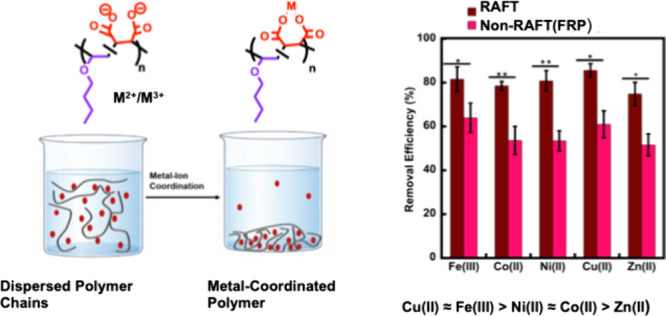

Water pollution is a major global challenge that affects ecosystems, public health, and sustainable development.^1^ Among various water contaminants, heavy metal ions are of particular concern because they are nonbiodegradable, persistent in aquatic environments, and capable of bioaccumulation in living organisms, even at low concentrations.^2,3^ Certain industrial activities significantly contribute to heavy metal pollution, highlighting the need for effective water remediation strategies.^4,5^

Conventional water treatment methods, including filtration, sedimentation, coagulation, flocculation, ion exchange, and advanced oxidation processes, are widely employed to remove contaminants.^6–8^ However, these methods often suffer from limitations, including high costs, operational complexity, challenges with recyclability, the production of harmful byproducts, and the need for additional resources and chemicals.^9–11^ Adsorption has therefore emerged as one of the most attractive approaches for water purification due to its simplicity, high efficiency, operational flexibility, and ability to remove contaminants at low concentrations.^12–14^ A wide range of adsorbents, including zeolites,^15^ silica,^16^ activated carbon,^17^ and graphene oxide,^18^ have been explored for heavy metal removal. Activated carbon (charcoal), one of the most widely used adsorbents, relies primarily on nonspecific interactions and physical adsorption.^19^ It often exhibits limited selectivity, reduced efficiency in multicomponent systems, and high energy requirements for regeneration.^9,12^

Polymer-based adsorbents have gained significant attention for water treatment because their chemical functionality and architecture can be tailored to promote selective interactions with target contaminants,^20^ while their tunable surface chemistry enables multiple removal mechanisms within a single platform.^21,22^ In particular, polymers containing carboxylic acid groups are promising because carboxylate ions can coordinate and bind metal cations.^23,24^ Despite these advantages, systematic studies evaluating polymeric adsorbents under conditions relevant to practical applications, including regeneration, competitive adsorption, equilibrium behavior, and continuous-flow operation, remain limited.

Vinyl ether maleic anhydride (VEMA) copolymers represent a promising platform for adsorption-based water treatment. VEMA exhibits a nearly alternating copolymer structure that provides a relatively uniform distribution of functional groups along the polymer backbone.^25,26^ This regular alternation provides a consistent spatial arrangement of dicarboxylate anion binding sites, which can promote more uniform and predictable metal–polymer interactions compared to a randomly functionalized polymer. The amphiphilic nature of the copolymer allows the hydrophobic vinyl ether units to modulate solubility, while the hydrophilic maleic anhydride units serve as reactive sites. Upon hydrolysis, the maleic anhydride groups in VEMA turn into carboxylate groups (COO^–^).^27^ These carboxylate groups can interact with metal cations via electrostatic attraction and coordination,^28,29^ enabling effective metal-ion capture (Scheme 1). The combination of a near-uniform distribution of functional groups, retention of polymer molar mass after adsorption, and reversible metal binding makes hydrolyzed VEMA particularly attractive for adsorption-based water treatment applications, where both performance and reusability are critical.

**Scheme 1:**
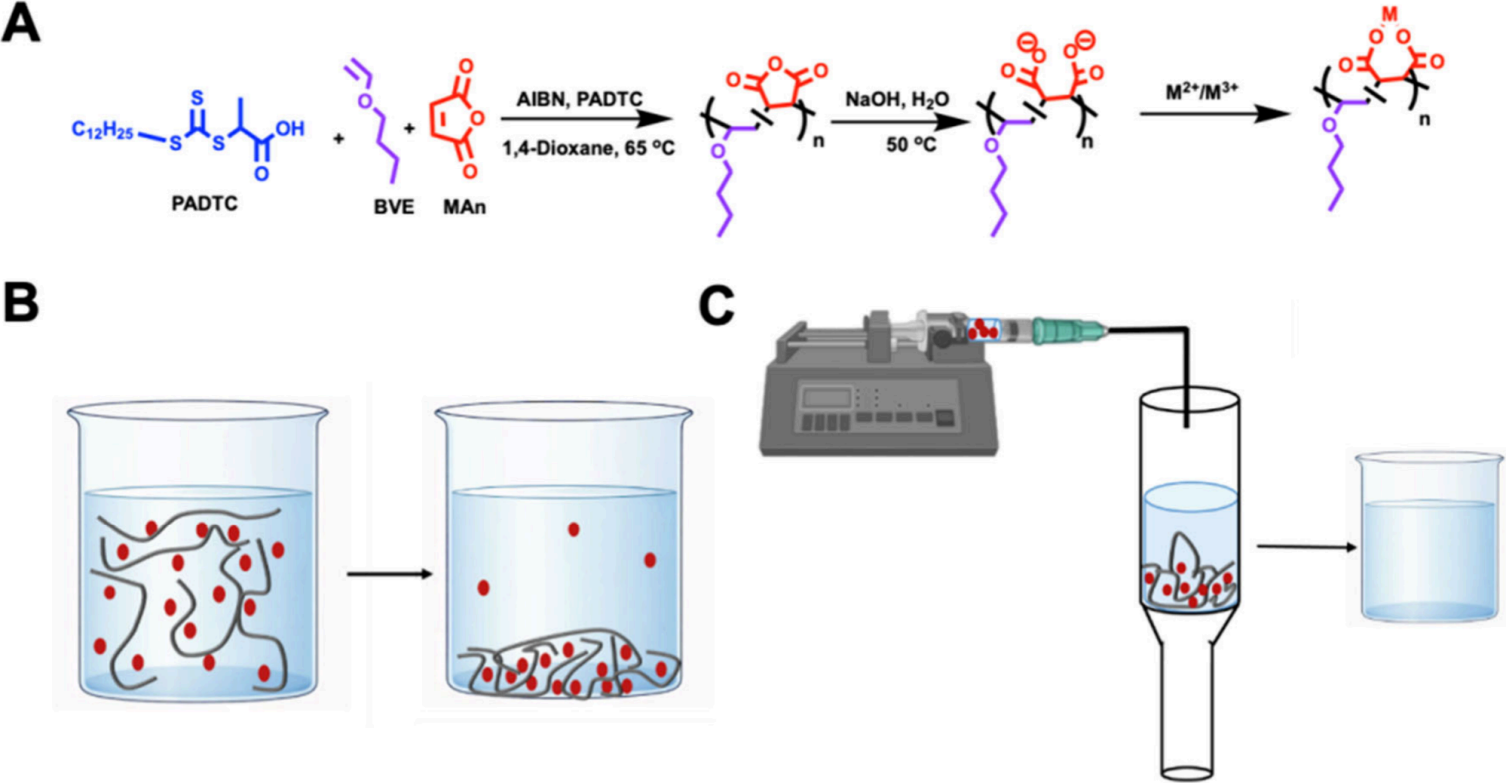
Schematic Illustration of (A) Synthesis, Hydrolysis, and Metal-Ion Coordination of VEMA Copolymer, (B) Batch Adsorption, and (C) Continuous-flow Adsorption

In this study, RAFT-synthesized VEMA polymers were examined for the adsorption of Fe­(III), Co­(II), Ni­(II), Cu­(II), and Zn­(II) ions from water. Adsorption performance was systematically evaluated under different pH and concentration conditions, as well as in competitive multimetal systems. In addition to adsorption kinetics, equilibrium isotherms, regeneration behavior, a comparison with activated carbon, and continuous-flow performance were examined to assess both the adsorption mechanism and practical applicability of VEMA-based polymer adsorbents for water remediation.

A VEMA copolymer was synthesized by RAFT copolymerization and subsequently hydrolyzed to generate carboxylate functionalities for metal ion binding. The molecular weight and hydrolysis were confirmed by Gel Permeation Chromatography (GPC) (Figure S2) and Fourier Transform Infrared (FT-IR) (Figure S3), respectively. For comparison, free radical polymerized VEMA (FRP-VEMA) was also synthesized and exhibited a higher apparent molar mass and broader dispersity than RAFT-VEMA, consistent with reduced polymerization control (Figure S2).

To evaluate the adsorption capability of the hydrolyzed VEMA copolymer toward metal ions, single metal adsorption experiments were initially conducted using Fe­(III), Co­(II), Ni­(II), Cu­(II), and Zn­(II) under identical experimental conditions, and the corresponding removal efficiencies are summarized in Figure 1A. Among tested metal ions, Cu­(II) showed the highest removal (86 ± 3%), followed by Fe­(III) (82 ± 6%), Ni­(II) (81 ± 5%) Co­(II) (79 ± 2%), and Zn­(II) (75 ± 5%). The consistently high adsorption efficiencies observed across all metals indicate a strong affinity of the hydrolyzed copolymer toward both divalent and trivalent metal cations. This adsorption behavior is primarily attributed to the carboxylate functionalities formed during the hydrolysis of maleic anhydride, which provide multiple coordination sites for metal binding through electrostatic attraction and coordination. The superior adsorption efficiency of Cu­(II) is consistent with its tendency to form stable complexes with oxygen-donor groups such as carboxylates.^30^ Similarly, the high adsorption efficiency observed for Fe­(III) is likely due to its high charge density and strong electrostatic interaction with the negatively charged polymer framework. Ni­(II) and Co­(II) exhibited comparable adsorption behavior, which is consistent with their similar ionic radii and coordination characteristics.^31^ At the same time, Zn­(II) showed comparatively lower removal efficiency, possibly because of its stronger hydration shell and weaker complexation tendency,^32,33^ relative to Cu­(II) and Fe­(III).

**Figure 1:**
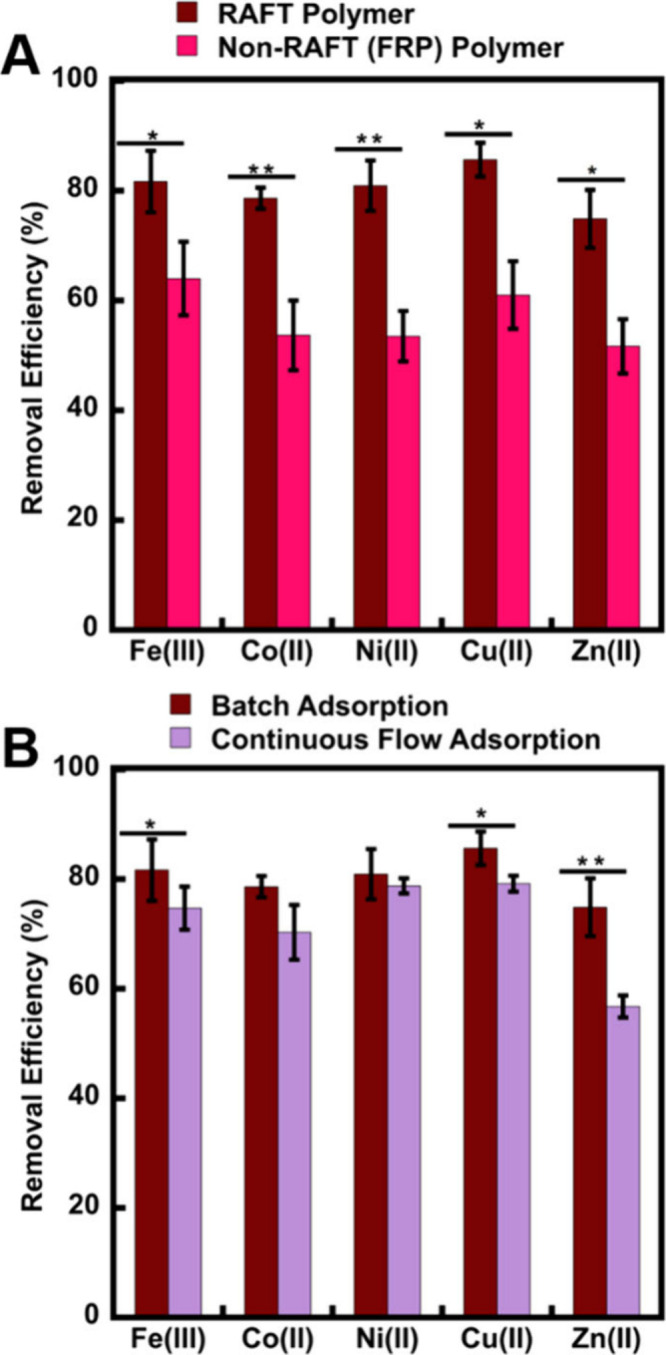
Removal efficiencies of Fe­(III), Co­(II), Ni­(II), Cu­(II), and Zn­(II) ions by VEMA copolymers: (A) RAFT and non-RAFT copolymers under batch adsorption conditions; (B) RAFT copolymer under batch and continuous-flow adsorption conditions (*p < 0.05, **p < 0.01).

To evaluate the influence of the polymerization method on adsorption performance, a non-RAFT (FRP) analogue was synthesized and evaluated under identical experimental conditions. Compared with the RAFT-synthesized polymer, the non-RAFT system exhibited consistently lower adsorption efficiencies for all investigated metal ions, with overall removal efficiencies reduced by approximately 15–25%. While the relative affinity trend remained similar, the lower performance may arise from the broader molar mass distribution and less controlled polymer architecture of the non-RAFT material, which could reduce the uniformity and accessibility of carboxylate binding environments and limit the formation of a stable metal cross-linked polymer phase.

To evaluate the practical applicability of the material under dynamic conditions, continuous-flow adsorption experiments were conducted in a single metal system (Figure 1B). Under continuous-flow conditions, the polymer effectively removed all investigated metal ions, with removal efficiencies ranging from 56% to 81%. Cu­(II) exhibited the highest removal (81 ± 2%), followed by Ni­(II) (78 ± 2%), Fe­(III) (75 ± 4%), Co­(II) (68 ± 8%), and Zn­(II) (57 ± 2%). The adsorption trend observed under flow conditions remained consistent with that observed in the batch adsorption experiments, further supporting the strong affinity toward Cu­(II). Slightly lower removal efficiencies under continuous-flow conditions are expected because of the reduced contact between the metal ion solution and the polymer.

To assess the influence of common background ions, adsorption was evaluated in the presence of Na^+^ and Ca^2+^ as monovalent and divalent electrolytes under both batch and continuous-flow conditions (Figure S5). Relative to the no-electrolyte control, adsorption effeicincies varied by up to approximately 20% in the presence of Na^+^ and up to approximately 30% in the presence of Ca^2+^, with increasing electrolyte concentration (1, 3, 10 mM) across the tested conditions for both batch and continuous-flow conditions. These results show that RAFT-VEMA retained adsorption performance in the presence of common background ions, while modest changes in efficiency likely occurred through partial competition with target metal ions for carboxylate-rich binding sites.

The adsorption kinetics of Fe­(III), Co­(II), Ni­(II), Cu­(II), and Zn­(II) onto the copolymer were subsequently analyzed to better understand the rate and mechanism of metal uptake (Figure S6) under batch conditions. Rapid adsorption was observed during the initial 12–24 h, followed by gradual equilibration after approximately 48 h, indicating fast initial surface coordination followed by progressive saturation of available active sites. The experimental data were well described by a reversible first-order kinetic model, with regression coefficients (R^2^) exceeding 0.96 for all investigated metal ions (Figure S7 and Table S1). Among the studied ions, Cu­(II) exhibited the highest adsorption capacity, further confirming its strong affinity toward the carboxylate-rich polymer network. Detailed kinetic parameters are provided in the Supporting Information.

The effect of solution pH on metal ion adsorption was evaluated over the pH range 2–10 (Figure 2A) under batch conditions. Adsorption efficiencies were low under strongly acidic conditions because protonation of carboxylate groups reduced interactions between the polymer and metal ions. Adsorption increased significantly from pH 4 to 6 due to progressive deprotonation, generating negatively charged carboxylate sites that enhanced metal coordination. Maximum adsorption was observed near pH 6 for all investigated metal ions, with Cu­(II) consistently exhibiting the highest removal efficiency. At pH values above 6, changes in metal speciation, including the formation of hydroxo complexes and possible hydroxide precipitation,^34^ may reduce the concentration of freely solvated metal ions available for direct coordination with the polymer, leading to lower apparent polymer-associated adsorption.

**Figure 2:**
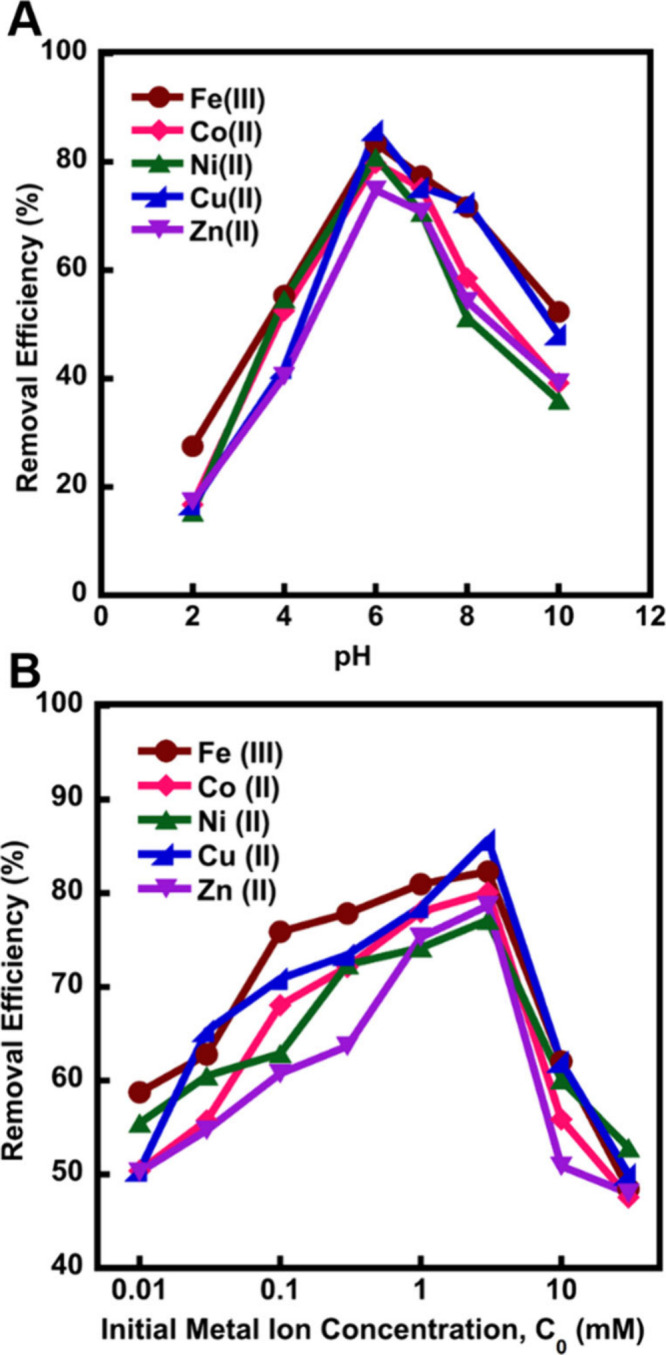
(A) Effect of pH on the adsorption efficiency of metal ions onto the polymer; (B) Effect of initial concentration on the adsorption efficiency of metal ions onto the polymer

The effect of initial metal ion concentration on adsorption efficiency was further evaluated over the concentration range of 0.01–30 mM (Figure 2B) under batch conditions. The removal efficiency initially increased with metal concentration, reaching a maximum around 1–3 mM, then gradually decreased at higher concentrations. At very low metal concentrations, the lower apparent removal may reflect a reduced concentration driving force for adsorption and limited formation of metal-mediated cross-links within the polymer-rich phase.^35,36^ Similarly, at higher concentrations, the adsorption efficiency was decreased, primarily attributed to the saturation of available active sites on the polymer surface and a reduction in the total number of cross-links relative to the total metal present.

The adsorption equilibrium behavior of Fe­(III), Co­(II), Ni­(II), Cu­(II), and Zn­(II) ions on the copolymer was analyzed using both the Langmuir and the Freundlich isotherm models. The Langmuir model assumes adsorption onto a uniform surface with a finite monolayer,^37^ whereas the Freundlich model accounts for adsorption on heterogeneous surfaces with nonequivalent adsorption sites.^38^ For the hydrolyzed VEMA copolymer, the carboxylate groups are chemically similar but may have different accessibility within the swollen polymer network. Consistent with this interpretation, the Freundlich model provided a better fit for most metal ions and a more physical description of adsorption within the heterogeneous hydrolyzed VEMA polymer network (Figure S8 and Table S2). The calculated 1/*n* values were below unity for all ions, suggesting favorable adsorption on a moderately heterogeneous surface. These results suggest that adsorption on the VEMA copolymer occurs through heterogeneous and reversible interactions distributed across multiple carboxylate binding sites.

Having established the adsorption capability of the VEMA copolymer toward individual metal ions, a more challenging competitive adsorption experiment was subsequently performed using a mixed metal system containing equal initial concentrations (3 mM) of Fe­(III), Co­(II), Ni­(II), Cu­(II), and Zn­(II) ions (Figure 3). Compared with the single metal adsorption experiments, lower removal efficiencies were observed because multiple ions competed simultaneously for the same adsorption sites. Despite the competitive environment, the polymer maintained clear selectivity toward Cu­(II), followed by Fe­(III), Ni­(II), Co­(II), and Zn­(II). Among the investigated ions, Cu­(II) exhibited the highest removal efficiency (78 ± 4%), demonstrating its strong affinity toward the carboxylate-rich copolymer even in the presence of competing metal ions. In contrast, Zn­(II) showed the greatest decrease in adsorption efficiency under competitive conditions, suggesting comparatively weaker coordination interactions with the polymer network.

**Figure 3:**
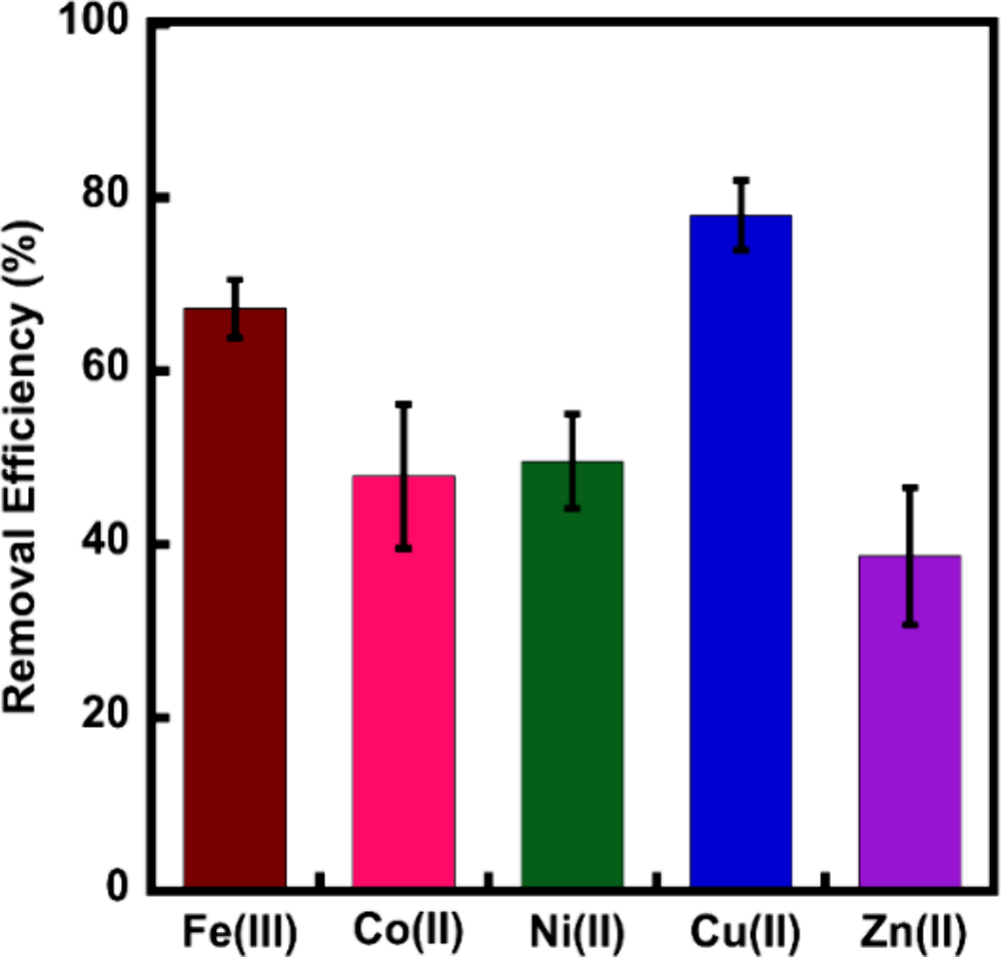
Removal efficiency of Fe­(III), Co­(II), Ni­(II), Cu­(II), and Zn­(II) by VEMA copolymer in a multimetal system.

The adsorption performance of the copolymer was further compared with that of activated carbon under identical experimental conditions (Figure S9). The copolymer exhibited higher removal efficiencies for Fe­(III), Ni­(II), and Cu­(II), whereas activated carbon showed slightly improved adsorption toward Co­(II) and Zn­(II). Among the investigated ions, Cu­(II) exhibited the highest removal efficiency with the copolymer, consistent with its strong coordination affinity toward carboxylate ligands. Compared with activated carbon, whose performance is often governed largely by surface area, pore structure, and nonspecific surface interactions, the hydrolyzed copolymer contains carboxylate functionalities that provide specific coordination sites for metal binding.^39,40^

Finally, the reusability and regeneration capability of the copolymer were evaluated through three consecutive adsorption–desorption cycles (Figure 4). Removal efficiency refers to the percentage of a given metal removed by the polymer, while desorption refers to the amount of that metal returned to the aqueous phase upon subsequent acid treatment. High adsorption efficiencies were observed during the first cycle for all investigated metal ions, ranging from 73−87%. Moderate decreases in adsorption efficiency were observed after repeated regeneration cycles, with removal efficiencies of 55–70% after three cycles. Chelation-assisted regeneration using EDTA was also examined; however, quantitative polymer recovery was not obtained across all metal ion systems. Despite the gradual decrease in adsorption performance under acidic conditions, desorption efficiencies remained relatively high throughout the regeneration experiments, indicating effective release of metal ions. The polymer mass gradually decreased over repeated adsorption–desorption cycles (Table S3), likely due to physical handling loss and possible partial dissolution during washing and regeneration procedures. While metal ion removal was quantified from the solution phase by measuring the residual metal concentration in the supernatant using UV–vis spectroscopy and ICP-OES, the adsorption process is expected to occur within the swollen, carboxylate-rich polymer phase of hydrolyzed VEMA. In this hydrated state, metal ions can access and interact with distributed carboxylate binding sites throughout the polymer-rich phase, supporting the observed decrease in solution phase metal concentration. However, during desorption and regeneration, this swollen physical state may contribute to handling loss during washing, separation, and acidic treatment, consistent with the gradual polymer mass loss observed over cycles. To determine whether metal ion adsorption affected the polymer molar mass distribution, GPC analysis was performed before and after adsorption. The GPC traces were largely overlapping, indicating that adsorption did not substantially alter the polymer molecular weight distribution (Figure S10).

**Figure 4:**
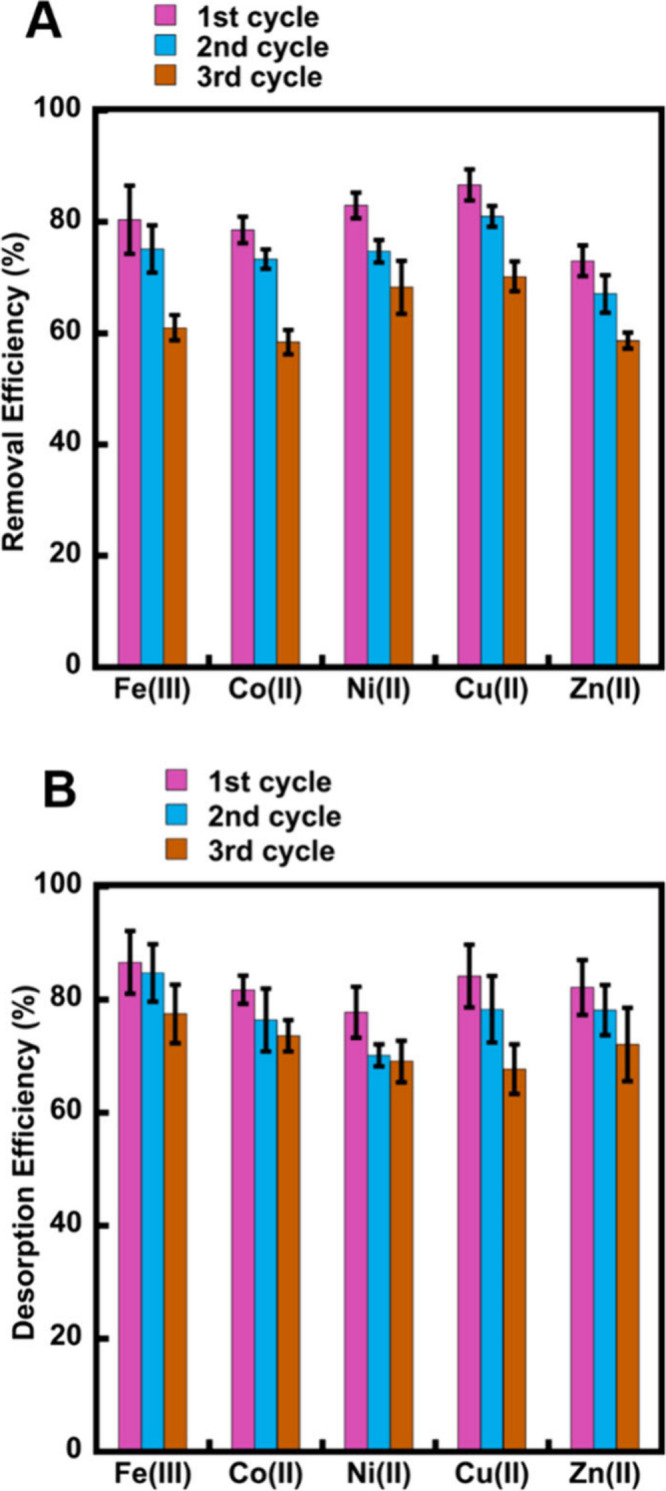
(A) Adsorption and (B) Desorption efficiencies of Fe­(III), Co­(II), Ni­(II), Cu­(II), and Zn­(II) ions on the polymer over three adsorption–desorption cycles.

The hydrolyzed VEMA copolymer demonstrated effective adsorption of Fe­(III), Co­(II), Ni­(II), Cu­(II), and Zn­(II) ions from water under batch, competitive, and continuous-flow conditions. The proposed mechanism for metal ion removal was primarily driven by electrostatic and coordination interactions between metal ions and the carboxylate functionalities, leading to the formation of a metal cross-linked hydrogel network. Among the metal ions, Cu­(II) and Fe­(III) consistently exhibited the highest adsorption efficiencies, reflecting their stronger affinity toward the carboxylate-rich polymer network. Comparison between RAFT and non-RAFT systems further demonstrated that controlled polymer architecture enhances adsorption performance. The adsorption behavior followed reversible first-order kinetics and was generally well described by the Freundlich isotherm model. In addition, the copolymer retained substantial adsorption capacity after multiple regeneration cycles, demonstrating its potential as a reusable adsorbent for heavy metal remediation applications.

## Supplementary Material



## Notes

Experimental section, supplemental NMR, GPC, IR, rheological and metal adsorption data (PDF)
