# Supplementary material for: Vinyl Ether Maleic Anhydride Copolymers: Efficient and Reusable Sorbents for Removing Heavy Metals from Water
Source: ACS Macro Lett. 2026 Jul 2;15(7):1035–41. doi: 10.1021/acsmacrolett.6c00265 (PMC13394388; doi:10.1021/acsmacrolett.6c00265)
Supplement: Supplementary file 1 [file mz6c00265_si_001.pdf]

Supporting Information: Vinyl Ether Maleic Anhydride Copolymers: Efficient and Reusable Sorbents for Removing Heavy Metals from Water

Ranjita Thapa Acharya, Muhammad Zeeshan Shah, Audrey Woodruff, Dominik Konkolewicz *

* Corresponding author: DK: d.konkolewicz@miamioh.edu

Department of Chemistry and Biochemistry, Miami University, 651 E High St, Oxford, OH, 45056, United States

1. Experiment

1.1 Materials and Methods

1.1.1 Reagents

Reagents for polymerization, butyl vinyl ether (BVE) and maleic anhydride (MAn), were purchased from Sigma-Aldrich and Chem-Impex International, Inc., respectively. The solvent 1,4-dioxane was supplied by TCI Chemicals, and sodium hydroxide (NaOH) pellets were purchased from Thermo Scientific. The radical initiator azobisisobutyronitrile (AIBN) from Sigma-Aldrich was stored at 4 °C before use. The chain transfer agent (CTA), 2-(propionic acid-yl dodecyl trithiocarbonate (PADTC), was synthesized according to the literature procedure.[1] The metal salts used for adsorption studies, including copper, zinc, iron, cobalt, and nickel chlorides, were purchased from Sigma-Aldrich, Thermo Scientific, MCIB, and J.T. Baker Chemicals, respectively. All reagents were used as received without further purification unless otherwise stated.

1.1.2 Instrumentation

A UV-visible spectrophotometer, an Atomic Absorption Spectrometer (AAS), and an Inductively Coupled Plasma-Optical Emission Spectrometer (ICP-OES) were used for quantitative metal ion analysis. The pH of the solution was measured using a calibrated digital pH meter. Fourier

transform infrared (FTIR) spectroscopy was used to analyze the polymer's hydrolysis. A Bruker 400 MHz spectrometer was used to monitor monomer conversion, and gel permeation chromatography (GPC) was used to determine the molecular weight of the synthesized polymer.

1.1.3 Metal Ion Solutions

Standard stock solutions (50 mM) of Fe(III), Co(II), Ni(II), Cu(II), and Zn(II) were prepared separately from their corresponding chloride salts in deionized water and diluted to the desired concentrations for adsorption studies.

1.2 Synthesis of VEMA Copolymer (RAFT Polymerization)

A typical RAFT copolymerization was carried out using a 66:50 molar feed ratio of butyl vinyl ether (BVE) and maleic anhydride (MAn), and AIBN was used at 30 mol% relative to CTA (PADTC). All reagents were dissolved in solvent 1,4-dioxane (monomer: solvent 1:2). After degassing with nitrogen for about 30 minutes, the reaction mixture was stirred in an oil bath at 65 °C for 4 h at 220 rpm. ^{1}H NMR and GPC analysis were used to determine monomer conversion and molecular weight, respectively.

1.2.1 Precipitation and Hydrolysis of VEMA Copolymer

The crude VEMA copolymer was purified by precipitation, following the procedure described in the literature.[2] A 100 mL beaker was filled with 60 mL of hexane and cooled in an ice bath for 5-10 minutes. The VEMA copolymer was added dropwise to the cold hexane, and the beaker was kept in an ice bath for several minutes to allow the polymer to precipitate. The supernatant was decanted, and the precipitate was dried in a vacuum desiccator for 24-48 h. After drying, deionized water (25 mL per g of polymer) was added, and 4 molar equivalents of aqueous NaOH were added dropwise. The flask was heated at 50 °C overnight for hydrolysis, and the hydrolyzed polymer was

precipitated and dried under vacuum for use in adsorption studies. FT-IR confirmed hydrolysis and indicated whether the maleic anhydride rings were open.

1.3 Batch Adsorption Studies

For adsorption studies, dry hydrolyzed polymer was used. Unless otherwise specified, batch adsorption experiments were carried out by mixing 0.1 g of polymer with 100 mL of a 3 mM metal ion solution. The pH of the solution was adjusted to 6 with 0.1 M HCl or NaOH as required. All samples were equilibrated at room temperature with continuous stirring at 220 rpm for 24 h. After equilibrium, the samples were centrifuged, and the supernatant was analyzed to determine the residual metal ion concentration. Metal ion concentrations before polymer addition and after adsorption were measured in all experiments, and metal ion removal (adsorption) efficiencies were calculated using the following equation (1).

$$\text{Removal efficiency (\%)} = \left(\frac{C_0 - C_e}{C_0}\right) \times 100 \tag{1S}$$

Where C_0 and C_e are the initial and final (equilibrium) metal concentrations (mM), respectively. Selected adsorption experiments were performed in triplicate, and the corresponding results are presented with error bars where applicable.

1.3.1 Single Metal Ion Adsorption (Individual Adsorption)

For a single-metal-ion adsorption experiment, each metal ion (Fe(III), Co(II), Ni(II), Cu(II), and Zn(II)) was independently equilibrated with the polymer under the batch conditions described above. After 24 h of equilibrium, the samples were centrifuged and analyzed.

1.3.2 Effect of pH

The effect of solution pH on adsorption was examined by adjusting the pH of metal ion solutions to 2, 4, 6, 7, 8, and 10 using 0.1 M HCl or NaOH. The solutions were equilibrated with the polymer

for 24 h under batch conditions, and the supernatant was collected and analyzed to determine residual metal ion concentrations.

1.3.3 Kinetic Study

Kinetic adsorption experiments were conducted to evaluate the rate of metal ion uptake by the polymer. For the kinetics study, samples were equilibrated, and aliquots were collected at specific time intervals (3, 6, 12, 24, 48 and 72 h). Each sample was centrifuged, and the supernatant was analyzed.

To account for simultaneous adsorption and desorption processes, the interaction of metal ions on the polymer surface was treated as a first-order reversible reaction,[3,4] which can be represented as:

$$A \underset{k_2}{\overset{k_1}{\leftrightharpoons}} B$$

Where A and B denote the metal ions in solution and those adsorbed on the polymer, respectively. The rate equation for the reversible process reaction is expressed as:

$$\frac{dC_B}{dt} = -\frac{dC_A}{dt} = k_1 C_A - k_2 C_B \qquad (2S)$$

Where C_A and C_B are the concentrations of metal ions in the solution phase and on the polymer, respectively, k_1 and k_2 are the forward (adsorption) and backward (desorption) first-order rate constants.

At equilibrium, the relationship between the rate constants is given by:

$$K_C = \frac{C_{Be}}{C_{Ae}} = \frac{k_1}{k_2} \qquad (3S)$$

Here, K_C is the equilibrium constant, and C_{Ae} and C_{Be} are the equilibrium concentrations of metal ions in the solution and the polymer, respectively.

Applying the equilibrium conditions in equation 2S, the integrated form of the rate equation[5] becomes:

$$\ln(1 - U_t) = -\,(k_1 + k_2)t \qquad (4S)$$

Where U_t is the fractional attainment of equilibrium, defined as:

$$U_t = \frac{C_{A0} - C_A}{C_{A0} - C_{Ae}} \qquad (5S)$$

Here, C_{A0} is the initial metal ion concentration, C_A is the concentration at time t, and C_{Ae} is the equilibrium concentration.

The equilibrium adsorption capacity (q_e) was calculated following the expression.[6]

$$q_e = \frac{(C_0 - C_e)V}{m} \qquad (6S)$$

Here, C_0 and C_e are the initial and equilibrium metal ion concentrations (mM), respectively, V is the solution volume (L), and m is the mass of the polymer (g). The values of q_e in mmol/g were converted into mg/g by using the corresponding atomic mass of each metal ion.

1.3.4 Adsorption isotherm

Adsorption isotherms were obtained by equilibrating the polymer with metal-ion solutions of varying initial concentrations (0.01, 0.03, 0.1, 0.3, 1, 3, 10, and 30 mM) for 24 h to ensure equilibrium. After equilibrium, the solutions were centrifuged, and the equilibrium metal ion concentrations were determined.

The equilibrium adsorption data were analyzed using the Freundlich isotherm model,[7] expressed in its linearized form as:

$$\ln(q_e) = lnK_F + \frac{1}{n}(lnC_e) \qquad (7S)$$

Where K_F is the Freundlich adsorption capacity constant, and n is the adsorption intensity parameter related to surface heterogeneity and the favorability of adsorption. The values of K_F and $\frac{1}{n}$ were determined from the linear plots of $\ln q_e$ versus $\ln C_e$.

Similarly, the Langmuir model[8] was also evaluated using its linearized form:

$$\frac{C_e}{q_e} = \frac{1}{q_{max}K_L} + \left(\frac{1}{q_{max}}\right)C_e \quad (8S)$$

Where q_{max} is the maximum monolayer adsorption capacity, and K_L is the Langmuir adsorption constant. The Langmuir model was evaluated to compare a homogeneous monolayer adsorption model with the Fréundlich heterogeneous adsorption model. The suitability of the two models was compared using the corresponding R^2 values.

1.3.5 Selective Adsorption (Multi-metal Adsorption)

For the selective adsorption study, 20 mL of each metal ion solution at an initial concentration of 3 mM was mixed. After 0.1 g of polymer was added, the mixture was equilibrated for 24 h. The initial and residual concentrations after centrifugation were quantified by ICP-OES.

1.3.6 Comparison with Activated Carbon

The adsorption performance of the polymer was compared with that of commercially available activated carbon. In these experiments, 0.1 g of each adsorbent was mixed with 100 mL of a 3 mM metal-ion solution for 24 h under identical conditions.

1.3.7 Desorption and Reusability

The recyclability of the polymer was evaluated through consecutive adsorption-desorption cycles to assess its regeneration capability and long-term stability. After each adsorption step, the polymer was separated by centrifugation, and the residual metal ion concentration in the supernatant was quantified. For desorption, the metal-loaded polymer was treated with 1 M HCl and equilibrated

for 24 h to facilitate the release of the adsorbed metal ions. The concentration of desorbed metal ions in the acid solution was subsequently analyzed. After desorption, the acid-treated polymer was thoroughly washed with deionized water to remove residual acid and then neutralized with 1 M NaOH. The regenerated polymer was subsequently reused for the next adsorption cycle under identical conditions. These adsorption-desorption cycles were repeated for three consecutive cycles to evaluate the reusability and stability of the polymer. The desorption efficiency was calculated using the following equation.[9]

$$\text{Desorption efficiency (\%)} = \frac{C_d V_d}{(C_0 - C_e)V_a} \times 100 \qquad (8)$$

Where C_d is the concentration of metal ions desorbed into the solution, V_d is the volume of desorption solution, C_0 and C_e are the initial and equilibrium metal ion concentrations during adsorption, respectively, and V_a is the volume of the initial adsorption solution.

1.4 Continuous-Flow Adsorption

In the continuous-flow experiment, 0.02 g of polymer was packed into a glass pipette supported by cotton. A syringe pump was used to continuously deliver 20 mL of a 3 mM metal-ion solution through the column at a constant flow rate. Effluent samples were collected and analyzed to assess adsorption performance under dynamic conditions.

1.5 Effect of Background Electrolyte

Background electrolyte experiments were performed under both batch and continuous flow conditions to evaluate the influence of common competing ions on RAFT-VEMA adsorption. The final concentration of each target metal ion was maintained at 3 mM. Na^+ and Ca^{2+} were used as representative monovalent and divalent electrolytes, respectively, at final concentrations of 1, 3, and 10 mM. A no-electrolyte sample was used as the control. After adsorption, the remaining metal

ion concentration was quantified, and the adsorption efficiency was calculated relative to the initial metal ion concentration. All experiments were performed in triplicate.

Result and Discussion

Structural Characterization of the VEMA Copolymer

A copolymer with a BVE: MAn molar feed ratio of 66:50 was synthesized to achieve near-complete and comparable conversion of both monomers. The polymer structure was confirmed by gel permeation chromatography (GPC), Fourier transform infrared (FT-IR) spectroscopy, and proton nuclear magnetic resonance (^{1}H NMR) spectroscopy. The ^{1}H NMR spectrum showed characteristic resonances of the VEMA copolymer (**Figure S1**), while GPC confirmed a narrower molar mass distribution for RAFT-VEMA (Mn ~ 13,000 g/mol with Mw/Mn = ~1.5) compared with free radical polymerized VEMA (FRP-VEMA), which exhibited a higher apparent molar mass and broader dispersity (Mn> 40,000 g/mol, Mw/Mn >3) (**Figure S2**).

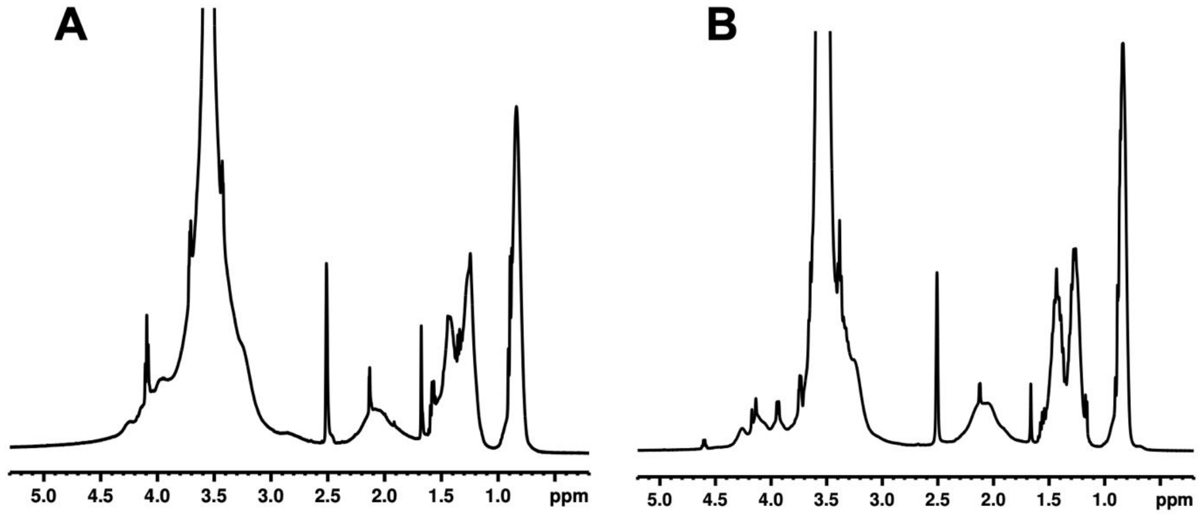

Figure S1: ^{1}H NMR spectra of A) RAFT-VEMA and B) Free Radical Polymerized VEMA (FRP-VEMA) copolymers in DMSO-d_6

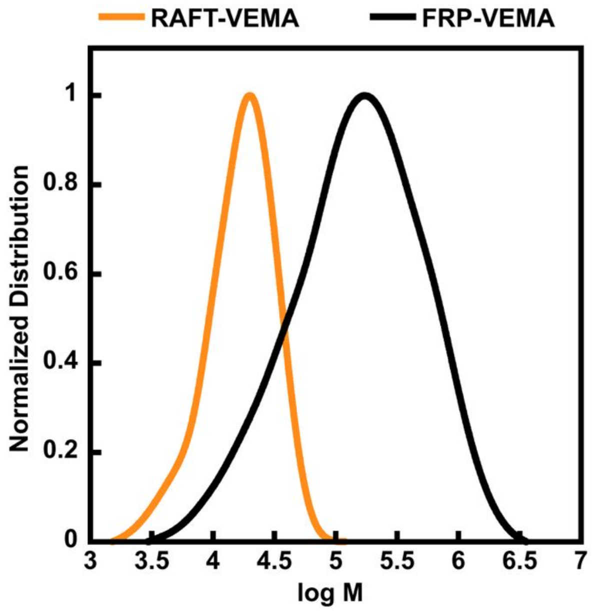

Figure S2: Normalized molecular weight distribution for VEMA copolymer synthesized via RAFT and FRP

The FT-IR spectrum (**Figure S3A**) displayed two characteristic anhydride carbonyl stretching bands at around 1858 cm^{-1} and 1779 cm^{-1}, confirming the incorporation of maleic anhydride into the polymer backbone. During hydrolysis, these anhydride peaks disappeared, and a carboxylate stretching band at 1600-1700 cm^{-1} appeared, indicating successful ring opening of the maleic anhydride groups consistent with reported values for maleic anhydride-containing polymers.[2] After interaction between metal ions and polymer (**Figure S3B**), shifts appeared at downshift around 1550-1650 cm^{-1}. These spectral shifts are consistent with metal ions binding to carboxylates via electrostatic interactions or chelation.[10] The backbone region (2800-3000 cm^{-1}, C-H stretching) remains unchanged before and after adsorption, demonstrating the polymer's structural integrity during metal uptake.

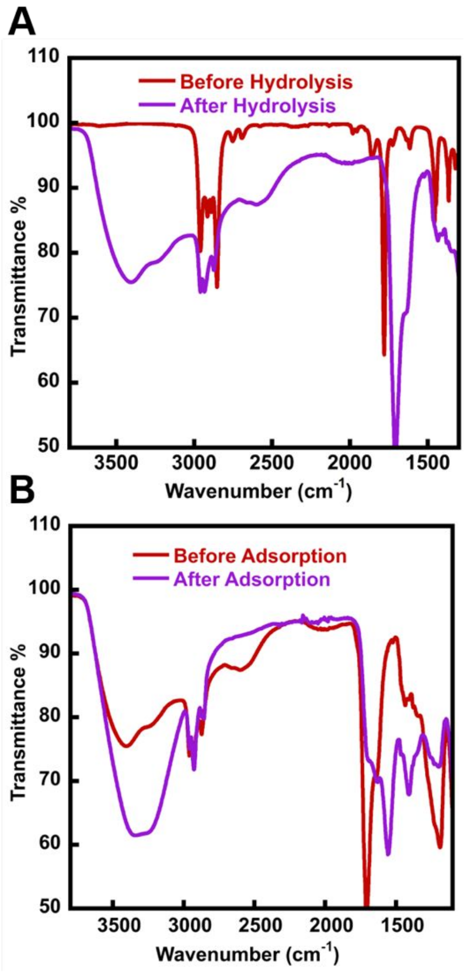

Figure S3: FTIR traces of VEMA copolymer **A**) Before and After Hydrolysis **B**) Before and After Adsorption

To further evaluate the physical state of the metal-loaded polymer after adsorption, oscillatory frequency sweep measurements were performed at 25 °C (**Figure S4**). Storage modulus (G′) and loss modulus (G″) were measured as a function of angular frequency for RAFT-VEMA after adsorption of Fe(III), Co(II), Ni(II), Cu(II), and Zn(II). The metal-loaded samples showed frequency-dependent viscoelastic behavior, with G′ comparable to or greater than G″ over much of the measured frequency range. These results indicate that the metal-loaded RAFT-VEMA samples retain a measurable viscoelastic response after adsorption, supporting the physical

stability of the polymer phase under the tested conditions.

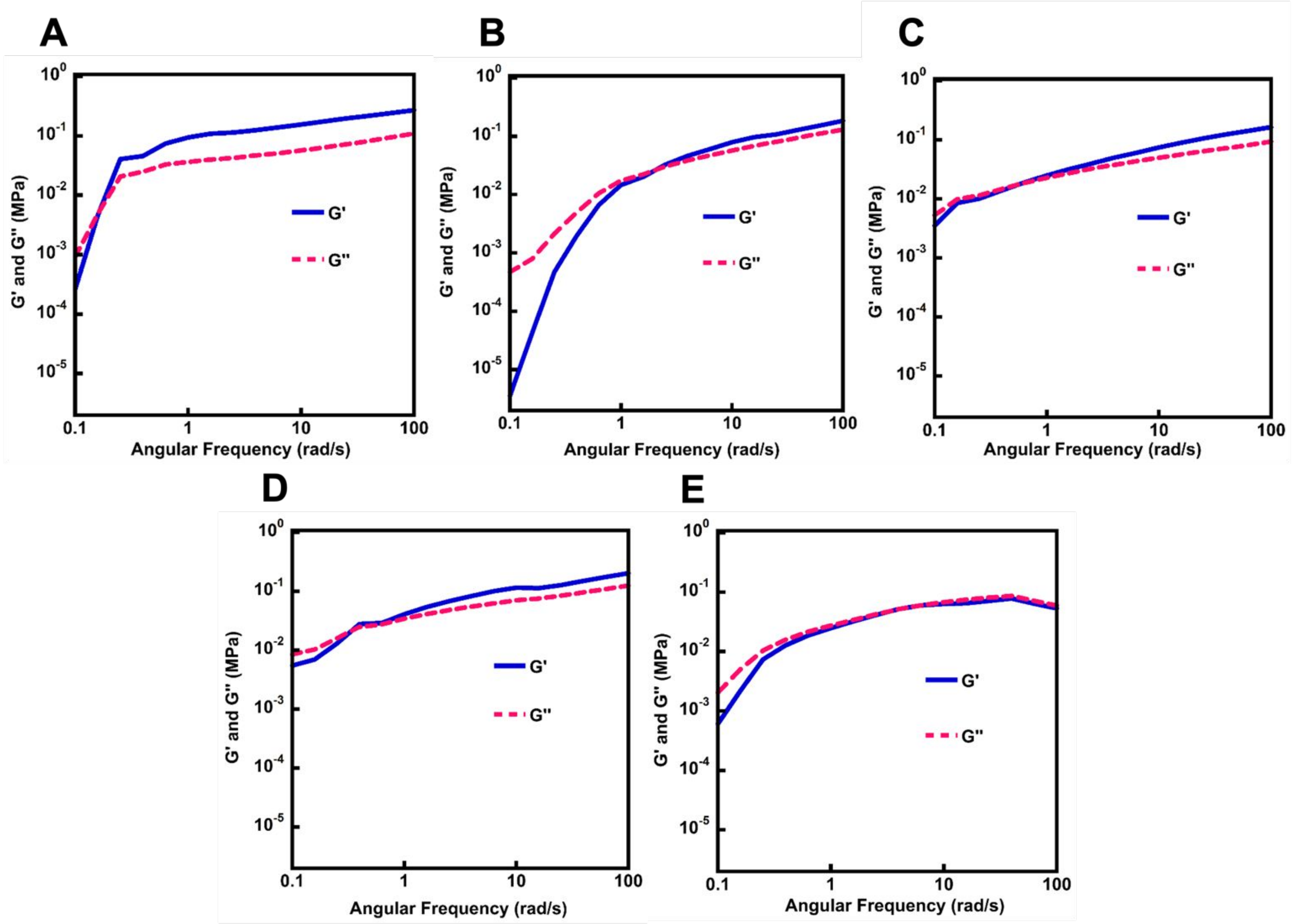

Figure S4: Oscillatory frequency sweep rheology of metal-loaded RAFT-VEMA after adsorption of A) Fe(III), B) Co(II), C) Ni(II), D) Cu(II), and E) Zn(II) at 25 °C. Storage modulus (G′) and loss modulus(G″) are plotted as a function of angular frequency.

Supplementary Data

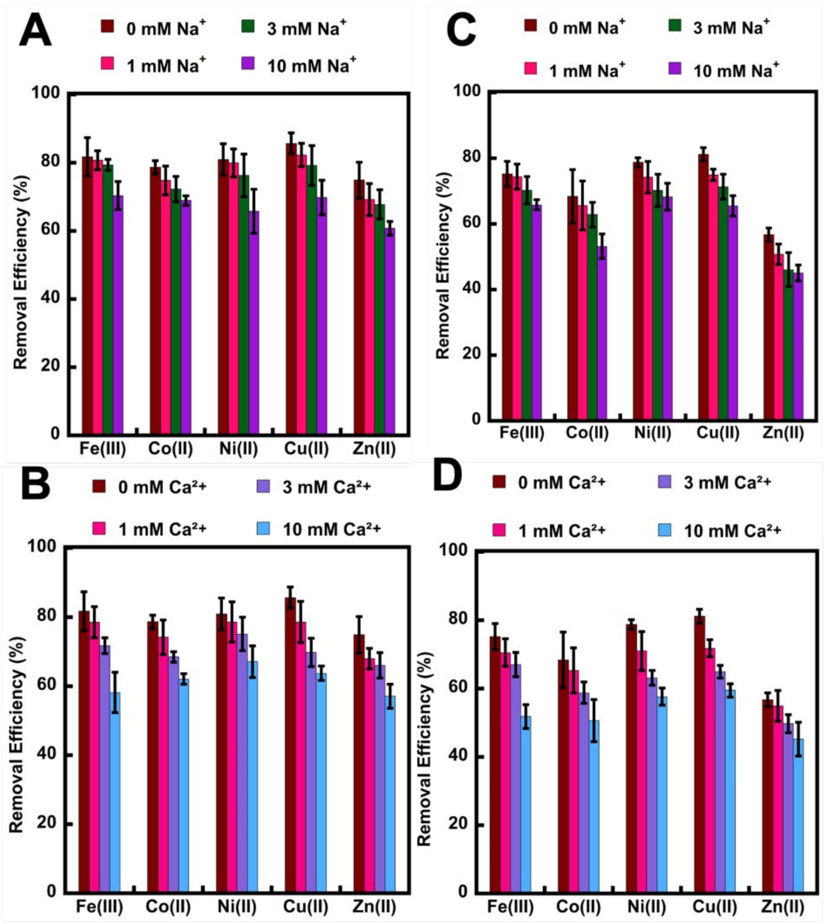

Figure S5: Effect of background electrolytes on metal ions by RAFT-VEMA under batch and continuous flow conditions. A) Batch adsorption in the presence of Na^{+}, B) Batch adsorption in the presence of Ca^{2+}, C) Continuous Flow adsorption in the presence of Na^{+}, D) Continuous Flow adsorption in the presence of Ca^{2+}

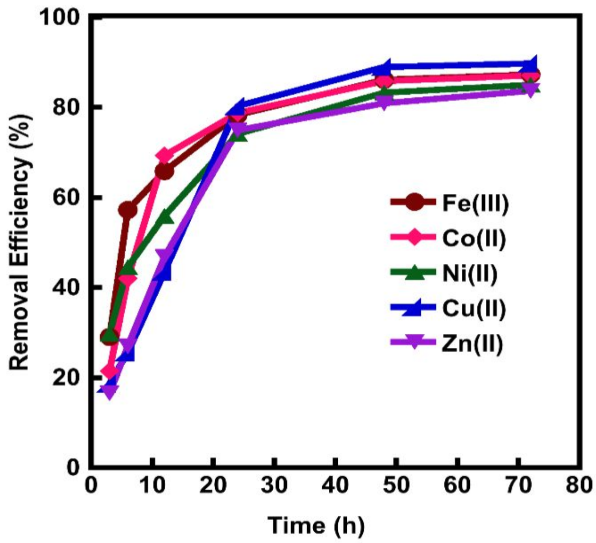

Figure S6: Time-dependent adsorption of metal ions onto the polymer

Table S1: Apparent Kinetic rate constants obtained from the reversible first-order adsorption model

Metal ions	k_1+k_2 (h^{-1})	q_e (mg/g)
Fe(III)	0.087	146.24
Co(II)	0.085	153.97
Ni(II)	0.076	149.70
Cu(II)	0.103	171.05
Zn(II)	0.074	164.11

Table S2: Freundlich isotherm parameters and Langmuir Fitting (R^2) for the adsorption of metal ions on the polymer.

Metal ions	Freundlich (n)	Freundlich (K_F)	Freundlich (R^2)	Langmuir (R^2)
Fe(III)	1.18	1.49	0.93	0.95
Co(II)	1.12	1.30	0.92	0.83
Ni(II)	1.09	1.38	0.96	0.80
Cu(II)	1.14	1.49	0.95	0.94
Zn(II)	1.12	1.12	0.94	0.77

Table S3: Recovered mass of polymer after three cycles

Metal ions	Initial (g)	1st cycle (Recovered) (g)	2nd cycle (Recovered) (g)	3rd cycle (Recovered) (g)
Fe(III)	0.100	0.094	0.086	0.078
Co(II)	0.100	0.091	0.081	0.071
Ni(II)	0.100	0.093	0.084	0.072
Cu(II)	0.100	0.092	0.083	0.075
Zn(II)	0.100	0.090	0.079	0.069

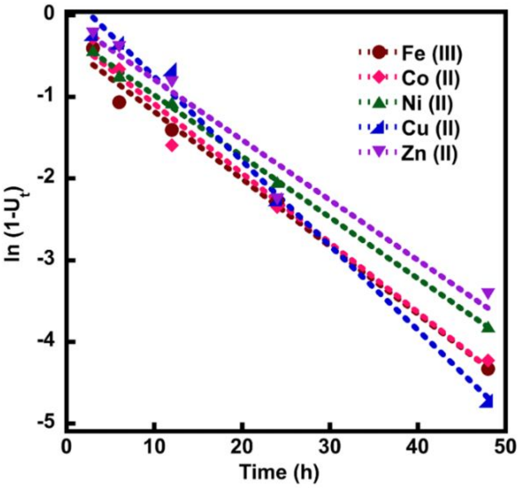

Figure S7: Linearized kinetic plots (ln(1-U_t) vs time) for the adsorption of metal ions onto the polymer

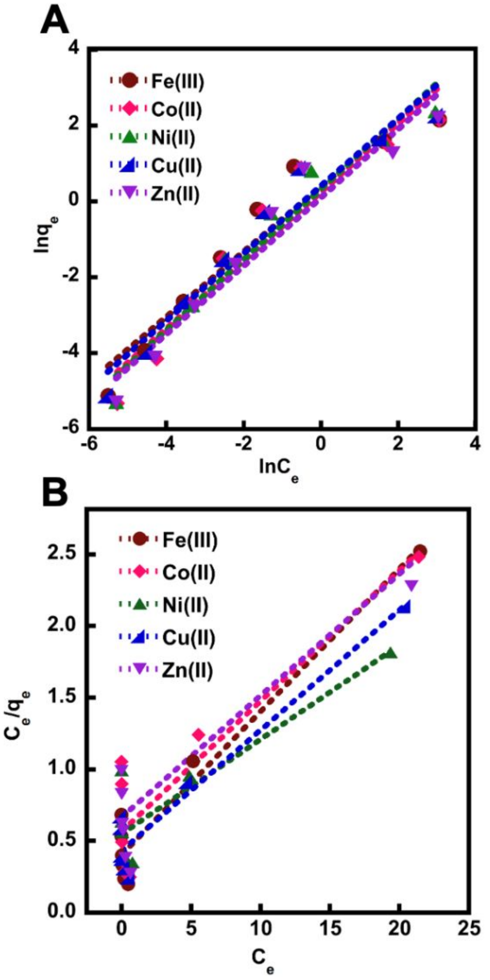

Figure S8: Adsorption isotherm fitting for Fe(III), Co(II), Ni(II), Cu(II), and Zn(II) adsorption on RAFT-VEMA **A)** Linearized Freundlich isotherm plots ($\ln q_e$ vs $\ln C_e$) for the adsorption of metal ions onto the polymer at equilibrium B) Linearized Langmuir isotherm plots for (C_e/q_e vs C_e) for the adsorption of metal ions onto the polymer at equilibrium.

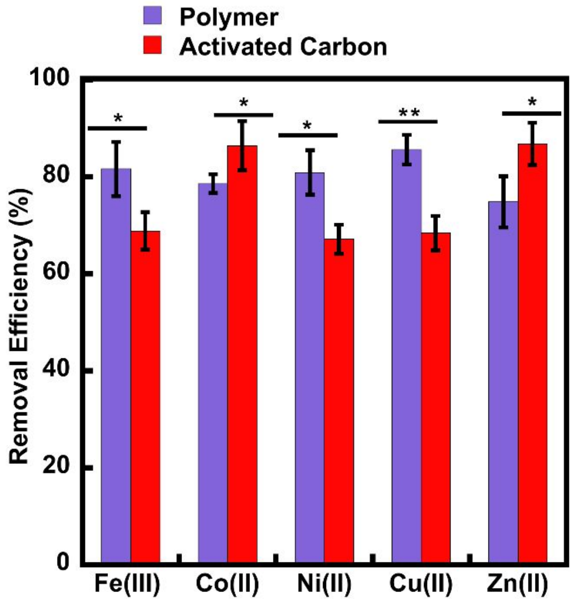

Figure S9: Comparison of metal ion removal efficiencies by the polymer and activated carbon. Statistical significance between two adsorbents is denoted by * ($p < 0.05$) and ** ($p < 0.01$).

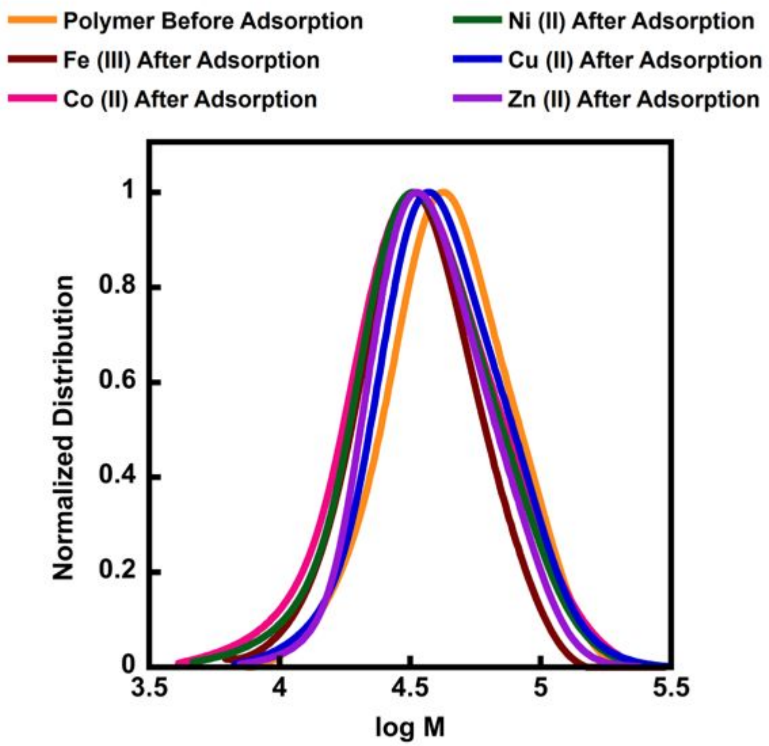

Figure S10: GPC traces of the RAFT-VEMA before and after adsorption of Fe(III), Co(II), Ni(II), Cu(II), and Zn(II).

References

(1) Craig, A. F.; Clark, E. E.; Sahu, I. D.; Zhang, R.; Frantz, N. D.; Al-Abdul-Wahid, M. S.; Dabney-Smith, C.; Konkolewicz, D.; Lorigan, G. A. Tuning the Size of Styrene-Maleic Acid Copolymer-Lipid Nanoparticles (SMALPs) Using RAFT Polymerization for Biophysical Studies. *Biochim. Biophys. Acta BBA - Biomembr.* **2016**, *1858* (11), 2931–2939. https://doi.org/10.1016/j.bbamem.2016.08.004.

(2) Shah, M. Z.; Okorafor, E.; Rotich, N. C.; Henoch, Q.; Thapa Acharya, R.; Page, R. C.; Lorigan, G. A.; Konkolewicz, D. Vinyl Ether Maleic Acid Block Copolymers: A Versatile Platform for Tunable Self-Assembled Lipid Nanodiscs and Membrane Protein Characterization. *Polym. Chem.* **2026**, 17 (2), 194-206. https://doi.org/10.1039/D5PY00767D.

(3) Bhattacharya, A. K.; Venkobachar, C. Removal of Cadmium(II) by Low Cost Adsorbents. *J. Environ. Eng.* **1984**, *110* (1), 110–122.http://dx.doi.org/10.1061/(ASCE)0733-9372(1984)110:1(110).

(4) Baral, S. S.; Das, S. N.; Rath, P. Hexavalent Chromium Removal from Aqueous Solution by Adsorption on Treated Sawdust. *Biochem. Eng. J.* **2006**, *31* (3), 216–222. https://doi.org/10.1016/j.bej.2006.08.003.

(5) Ho, Y. S.; McKay, G. Pseudo-Second Order Model for Sorption Processes. *Process Biochem.* **1999**, *34* (5), 451–465. https://doi.org/10.1016/S0032-9592(98)00112-5.
(6) Sadik, R.; Lahkale, R.; Hssaine, N.; ElHatimi, W.; Diouri, M.; Sabbar, E. Sulfate Removal from Wastewater by Mixed Oxide-LDH: Equilibrium, Kinetic and Thermodynamic Studies. J. *Mater. Environ. Sci.* **2015**, 6 (10), 2895-2905.
(7) Kalam, S.; Abu-Khamsin, S. A.; Kamal, M. S.; Patil, S. Surfactant Adsorption Isotherms: A Review. *ACS Omega* **2021**, *6* (48), 32342–32348. https://doi.org/10.1021/acsomega.1c04661.
(8) Langmuir, I. The adsorption of gases on plane surfaces of glass, mica and platinum. *J. Am. Chem. Soc.* **1918**, *40* (9), 1361–1403. https://doi.org/10.1021/ja02242a004.
(9) Rahman, Md. A.; Lamb, D.; Rahman, M. M.; Bahar, M. M.; Sanderson, P. Adsorption–Desorption Behavior of Arsenate Using Single and Binary Iron-Modified Biochars: Thermodynamics and Redox Transformation. *ACS Omega* **2022**, *7* (1), 101–117. https://doi.org/10.1021/acsomega.1c04129.
(10) Nara, M.; Tanokura, M. Infrared Spectroscopic Study of the Metal-Coordination Structures of Calcium-Binding Proteins. *Biochem. Biophys. Res. Commun.* **2008**, *369* (1), 225–239. https://doi.org/10.1016/j.bbrc.2007.11.188.
